# Benefits of forest therapy for adult mental health: a systematic review and meta-analysis based on the Profile of Mood States (POMS)

**DOI:** 10.3389/fpsyg.2025.1670804

**Published:** 2025-11-03

**Authors:** Xueyan Shang, Jiahao Lu, Meng Tao, Cheng Fei, Jiaming Fei

**Affiliations:** ^1^School of Physical Education, Zhejiang Guangsha Vocational and Technical University of Construction, Jinhua, China; ^2^Taizhou Vocational College of Science and Technology, Taizhou, China; ^3^School of Exercise and Health, Shanghai University of Sport, Shanghai, China; ^4^Department of Cultural Management, Moscow State University, Moscow, Russia; ^5^School of Physical Education, Huainan Normal University, Huainan, China

**Keywords:** forest therapy, adults, mental health, meta-analysis, physical activity

## Abstract

**Objective:**

This study aims to assess the effects of forest therapy on human mental health through meta-analytic methods and to examine the moderating variables that influence this relationship. The goal is to provide a scientific basis for optimizing forest therapy interventions tailored to different populations to enhance mental health outcomes.

**Methods:**

Databases such as CNKI, Wanfang, Web of Science, PubMed, Cochrane, and Embase were utilized for data collection, and data processing was performed using EndNote X9 and Stata 16.0 statistical software. The weighted mean difference (WMD) and 95% confidence interval (CI) were used as effect size indicators for the meta-analysis, and relevant moderator variables were tested.

**Results:**

The study analyzed three subgroups based on intervention duration, exercise intensity, and participant origin. Subgroup 1 (*t* ≤ 15 min), Subgroup 2 (15 min < *t* < 60 min), and Subgroup 3 (*t* ≥ 60 min) were categorized by intervention duration. Participants were further divided into Static forest therapy (Subgroup 1) and Dynamic forest therapy (Subgroup 2) based on exercise intensity. Additionally, participants were classified as either Asian (Subgroup 1) or European (Subgroup 2) based on their origin. The results indicated that longer single sessions of forest therapy were more beneficial in improving both positive and negative psychological states. Dynamic forest therapy was more effective than static forest therapy in enhancing these states. Furthermore, forest therapy was found to be more effective in improving both negative emotions (e.g., nervousness, depression, confusion) and positive emotions (e.g., vitality) in Asian populations compared to European populations.

**Conclusion:**

Forest therapy has been shown to effectively alleviate anxiety, anger, depression, fatigue, and confusion, while also enhancing vitality. However, when the duration of the therapy is less than one hour, its effects on reducing fatigue and enhancing vitality are less pronounced.

## 1 Introduction

Urbanization has accelerated the shift of residents’ living spaces from natural environments to built environments, providing convenience and comfort for urban dwellers. However, this transformation has also led to increased psychological pressure due to the fast-paced lifestyle (Kabisch et al., 2021; Levin et al., 2021). Concurrently, chronic health issues resulting from environmental pollution and prolonged exposure to ozone have posed significant challenges for urban residents (Yuan et al., 2023). Numerous studies have shown that seeking ecological exposure in natural environments is a scientifically valid strategy for promoting health (Dzhambov et al., 2024; Hossain et al., 2020; Zhu et al., 2021). Physical exercise in natural environments has been found to improve mental health conditions such as depression and anxiety (Lubans et al., 2016; Singh et al., 2023). Forest therapy, integrating ecological exposure, physical activity, health promotion, and positive mindfulness meditation, finds its theoretical foundation in the Green-Blue Movement theory—which posits that the combined benefits of physical activity in natural settings far exceed the sum of similar activities in artificial environments (Pretty et al., 2005). Forest therapy serves as an effective intervention for stress relief, utilizing various natural elements to stimulate the senses (sight, smell, and hearing) and enhance the immune system, thereby promoting overall physiological health (Oh et al., 2017; Stobbe et al., 2024).

Previous studies have shown that forest therapy positively affects physiological and biochemical factors such as blood pressure, lung function, salivary cortisol, brain waves, and lymphocyte levels (Barbieri et al., 2020; Lee et al., 2014). Mental health improvements have also garnered significant attention, particularly as urban populations grow and chronic exposure to stressful environments increases the risk of mental health problems (Kabisch et al., 2021). Forest walking has been found to reduce depression and anxiety, promote positive thinking (Ameli et al., 2021; Miyazaki et al., 2014), and significantly enhance mood and quality of life (Bang et al., 2016). Intervention programs combining virtual reality (VR) and video have also proven effective in improving mood, alleviating depression, and fostering a sense of recovery (Poli et al., 2024).

Recent meta-analyses and systematic reviews have primarily examined the effects of forest therapy on physiological indicators such as immune function and salivary cortisol (Oh et al., 2017), as well as its overall impact on psychological indicators like depression and anxiety (Hossain et al., 2020). However, variations in the psychological benefits of forest therapy across different intervention durations, populations, and between dynamic and static therapies remain under-explored (Bang et al., 2016). This study, based on randomized controlled trials, explores the psychological benefits of varying intervention durations, dynamic and static forest therapy, and their effects across different ethnic groups through subgroup analysis. It also investigates the impact of forest therapy on subjective mental health indicators and physiological health parameters. Furthermore, the study identifies and summarizes moderating variables related to specific populations and forest systems, analyzing the results in light of subgroup variations and the current development of forest therapy. The findings provide theoretical insights and a scientific basis for the development, social integration, and application of forest therapy.

## 2 Materials and methods

### 2.1 Prospero register

PROSPERO is a prospective systematic review registration system developed by the Centre for Reviews and Dissemination (CRD) at the National Institute for Health Research (NIHR) in the United Kingdom. Its purpose is to ensure the objectivity and transparency of non-Cochrane systematic reviews and to provide robust evidence for evidence-based decision-making. Registration for this study has been completed on the PROSPERO website (NO.CRD42025631042).

### 2.2 Literature search

The following English search terms were used: “Forest Health,” “Forest Bathing,” “Forest Healing,” “Forest therapy,” “mental health,” “psychology,” “POMS,” “depression,” “stress,” “emotion,” “Randomized,” and The search strategy was developed based on the characteristics of each database, employing a combination of subject terms and free-text words. The databases searched included CNKI, Wanfang, Web of Science, PubMed, Cochrane, and Embase, totaling six databases. The literature review spanned from the inception of the databases to June 2024.

### 2.3 Literature inclusion and exclusion criteria

Based on the PICOS criteria in evidence-based medicine. PICOS is a standardized framework used in systematic reviews to structure clinical questions. Its five letters stand for: P (Population): Study subjects; I (Intervention): Intervention measures; C (Comparison): Control measures; O (Outcome): Outcome measures; S (Study design): Research design (Mehrdad and Ali, 2020). The inclusion criteria for the literature were as follows: (1) the population consisted of adults aged 18–75 years; (2) the intervention involved forest therapy, forest viewing, or other activities that facilitated contact with the forest environment (e.g., meditation, sitting, viewing, walking, or a combination of these activities); (3) the comparison group included indoor, urban, or other control groups that were distinct from the forest therapy intervention; (4) the outcome measures include the mood state scale, a widely used self-report psychological assessment tool designed to evaluate an individual’s transient emotional state, subjective assessments (e.g., depression, anxiety, happiness, vitality, recovery, etc.), or physiological and biochemical markers (e.g., blood pressure, heart rate, salivary cortisol, adrenaline, brain waves, etc.); and (5) the study design was a randomized controlled trial (RCT).

The following literature was excluded: (1) review articles, meta-analyses, and theoretical articles, as they constitute secondary research and cannot provide raw data for effect size pooling. (2) Studies with inconsistent interventions in the experimental or control groups, to ensure validity of intergroup comparisons and avoid bias in results due to confusion in intervention definitions. (3) Interventions combining forest therapy with medication or dietary supplements, as the goal is to assess the independent, pure effect of forest therapy rather than its combined effect with other treatments. (4) Studies with inconsistent research paradigms or outcome measures, to ensure methodological quality and comparability of outcome measurements across included studies, thereby enabling a valid meta-analysis. (5) Studies lacking a control group or key data (e.g., mean, standard deviation) that could not be obtained from the authors, as these are fundamental requirements for inter group comparisons and effect size calculations.

### 2.4 Data extraction

EndNote X9 software was used to select the literature. Based on the inclusion and exclusion criteria, two researchers independently screened the literature and reviewed the full text to determine eligibility for inclusion. Data were extracted from studies that met the inclusion criteria. Each researcher created separate Excel tables to extract relevant information, including the basic details (author, year, title) of the studies, information about the experimental and control groups (e.g., sample size, age, intervention measures, forest characteristics, outcome measures), and experimental design and quality evaluation data. The selected studies were then cross-checked, and any discrepancies in the screening results were resolved through discussion with a third researcher.

### 2.5 Statistical analysis

(1) Effect sizes were calculated using Stata 16 software. All outcome indicators in this study were continuous variables with the same unit of measurement. Thus, the weighted mean difference (WMD) and 95% confidence interval (CI) were used as effect size indicators. The following thresholds were applied to determine effect sizes: |WMD| < 0.2 was considered a small effect size, 0.2 ≤ |WMD| < 0.5 was a moderate effect size, 0.5 ≤ |WMD| < 0.8 was a medium effect size, and |WMD| ≥ 0.8 was a large effect size. Heterogeneity across studies was assessed using the Q test and the I^2^ statistic, both derived from the Stata 16 forest plot. Statistical significance was set at *p* < 0.05. A fixed-effect model was used for analysis when 0 < I^2^ < 50%, while a random-effects model was applied when 50% ≤ I^2^ < 75%. For I^2^ ≥ 75%, sensitivity analysis was conducted to explore the source of heterogeneity, followed by subgroup analysis based on the identified sources of heterogeneity.

(2) Stata 16.0 software was used to create a funnel plot and perform a publication bias test for this study. Since funnel plot interpretation is highly subjective, a quantitative test based on Egger’s method was conducted to assess publication bias. A *p* < 0.05 indicates the presence of publication bias.

## 3 Results

### 3.1 Screening results

**FIGURE 1:**
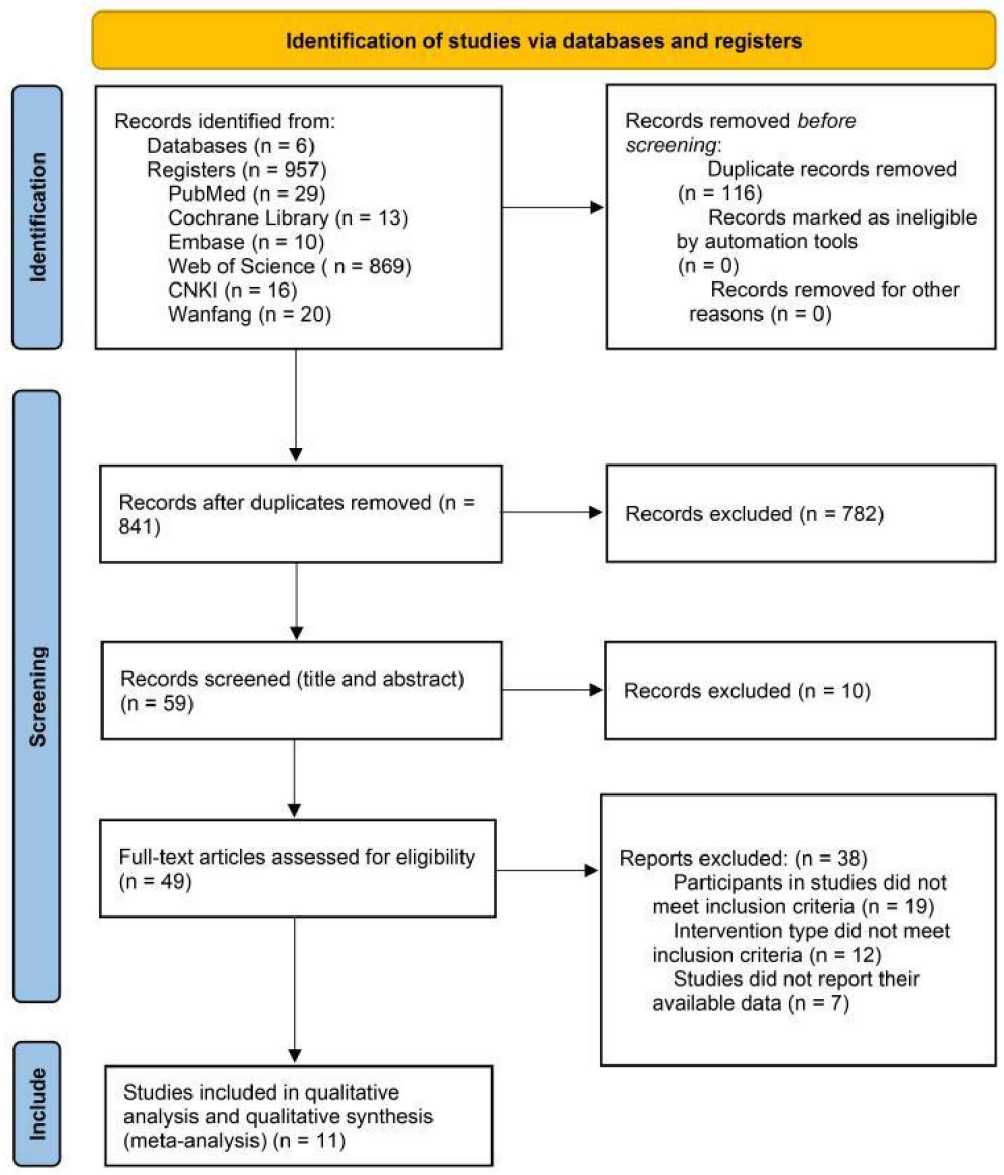
Flowchart of literature screening.

A total of 957 studies were initially screened. We first used the automatic duplicate detection feature in EndNote X9 software to remove 116 duplicated publications. Subsequently, two reviewers independently screened the titles and abstracts of the remaining 841 articles based on inclusion and exclusion criteria. At this stage, meta-analyses, review articles, and cross-sectional studies were further excluded via EndNote, leaving 59 articles to proceed to the next screening phase. In the second round, full-text screening identified studies lacking POMS scale scores or with inaccessible data, resulting in the inclusion of 11 studies in the meta-analysis (Figure 1).

### 3.2 Basic characteristics of literature

The meta-analysis included 15 experimental groups, comprising 418 participants (Table 1), and 13 control groups with 433 participants. All participants were adults aged 18 to 60 years. They were from three Asian countries (South Korea, Japan, and China) and three European countries (Poland, Finland, and Germany). The interventions and control conditions for forest therapy included indoor video experiences vs. no video, outdoor vs. indoor, forest vs. field, and forest vs. city. Janeczko et al. (2020) randomly assigned 75 participants to two sets of RCTs, with two forest and two urban environments. In the study by Liu et al. (2021), 30 participants were assigned to groups based on mixed forest, coniferous forest, broadleaf forest, and city environments, forming three RCT groups.

**TABLE 1:** Basic characteristics of literature.

References	*N* experimental/control	Age	Intervention	Indicators
Horiuchi et al., 2014	15 (15/15)	M = 36 SD = 8	Forest viewing vs. Indoor meditation (15 min); cross-over trial	T, D, A, F, C, V
Grebner, 2019	32 (16/16)	M = 20.97 SD = 0.65	Forest viewing vs. city viewing (15 min); RCT	T, D, A, F, C, V
Janeczko et al., 2020	75 (17/23; 13/22)	19∼24	Trial 1: Apartment suburb vs. coniferous forest; trial 2: green suburb vs. deciduous forest; walk 2 km (30 min); RCT	T, D, A, F, C, V
Bielinis et al., 2020	42 (42/42)	M = 26.24 SD = 6.23	Forest viewing vs. CityVideo (15 min); cross-over trial	T, D, F, V
Liu et al., 2021	30 (30/30; 30/30; 30/30)	22∼28	Trial 1: Mixed forest vs. city (70 min); Trial 2: Broad-leaved forest vs. city (70 min); Trial 3: Coniferous forest vs. city (70 min); cross-over trial	T, D, A, F, C, V
Katja et al., 2022	38 (23/23; 15/15)	M = 44.66 SD = 15.67	Summer: forest vs. field (60 min); Autumn: forest vs. field (60 min); cross-over trial	D, A, F, V
BumJin et al., 2022	53 (53/53)	40∼65	Forest vs. city (3 days); cross-over trial	T, D, A, F, C, V
Wen et al., 2023	20 (10/10)	Trial (M = 24.1 SD = 1.5); control (M = 23.7 SD = 1)	Forest vs. city viewing (20 min); RCT	T, D, A, F, C, V
Gun et al., 2021	38 (19/19)	M = 22.1 SD = 1.6	Forest therapy vs. daily activities (8 times); RCT	T, D, A, F, C, V
Takayama et al., 2019	46 (46/46)	M = 21.125	Forest walking vs. city walking (15 min); cross-over trial	T, D, A, F, C, V
Bielinis et al., 2018	62 (31/31)	M = 21.45 ± 0.18	Forest viewing vs. city viewing (15 min); RCT	T, D, A, F, C, V

T, tension and anxiety subscale; D, depression subscale; A, anger and hostility subscale; F, fatigue subscale; C, confusion subscale; V, vigor subscale.

### 3.3 Literature quality evaluation

A total of 15 randomized controlled trials (RCTs) were included across 11 studies (Table 2). Of these, only one study described the randomization method, while the remaining studies indicated randomization but did not provide a clear description of the method. Therefore, the “unclear” option was selected for the random sequence generation criterion. If the text indicated that informed consent was obtained, it was assumed that the participants were not blinded. Given the absence of significant dropout cases, the inclusion of indicators other than the POMS scale, and the comprehensive nature of the reported indicators, no evidence of selective reporting was found. As a result, the “low risk” option was selected for other evaluation criteria. The overall quality of the 11 studies was high.

**TABLE 2:** Literature quality evaluation.

Literature	Random	Distribution scheme	Blind method		Outcome indicator	Research result	Others	Evaluation
			Subject	Data processing				
Horiuchi et al., 2014	Low	Unclear	High	Unclear	Low	Low	Low	B
Grebner, 2019	Low	Low	High	Unclear	Low	Low	Low	B
Janeczko et al., 2020	Low	Unclear	High	Unclear	Low	Low	Low	B
Bielinis et al., 2020	Low	Low	High	Unclear	Low	Low	Low	B
Liu et al., 2021	Low	Unclear	High	Unclear	Low	Low	Low	B
Katja et al., 2022	Low	Unclear	High	Unclear	Low	Low	Low	B
BumJin et al., 2022	Low	Low	Low	Low	Low	Low	Low	A
Wen et al., 2023	Low	Unclear	High	Unclear	Low	Low	Low	B
Gun et al., 2021	Low	Low	Low	Unclear	Low	Low	Low	B
Takayama et al., 2019	Low	Unclear	High	Unclear	Low	Low	Low	B
Bielinis et al., 2018	Low	Unclear	High	Unclear	Low	Low	Low	B

### 3.4 Effect size combination and subgroup analysis

(1) Subgroup analysis of intervention duration

The results of the subgroup analysis and heterogeneity test based on different intervention durations are presented in Table 3. The T-scale score was used as the outcome variable. For subgroup 1, the combined effect size indicated that, compared to the control group, the experimental group showed reduced tension-anxiety mood; however, the effect size was not significant (*Z* = 1.00, *P* = 0.318), despite a large effect size (WMD = −2.304). In subgroup 2, the effect size of the experimental group was significantly lower than that of the control group, with a significant reduction in tension-anxiety mood (*Z* = 2.45, *P* = 0.014), corresponding to a medium effect size (WMD = −0.458). In subgroup 3, the experimental group showed a significant reduction in tension-anxiety mood compared to the control group (*Z* = 4.89, *P* = 0.000), with a large effect size (WMD = −1.794). Additionally, the effect size results for subscale scores of D, A, F, C, and V as outcome indicators all suggested that the experimental group exhibited greater mood improvement. However, the effect sizes for subgroup 1 in indicators D, A, and F were not significant. In subgroup 2, the effect sizes for indicators F, C, and V were not significant. By contrast, all indicators in subgroup 3 showed significant effect sizes. These results suggest that longer-duration forest therapy activities are more effective in improving both positive and negative psychological states, with specific effect size levels shown in Table 3.

**TABLE 3:** Subgroup analysis of intervention duration.

Outcome indicators	Subgroup 1: (*t* ≤ 15 min) 2: (15 min < *t* < 60 min) 3: (*t* ≥ 60 min)	Quantity	Heterogeneity test	Effect size	
			I^2^% (*P*)	WMD (95% CI)	Z (*P*)
T	1	2	90.2% (0.001)	−2.304 (−6.822∼2.215)	1.00 (0.318)
	2	3	67.6% (0.046)	−0.458 (−0.824∼−0.092)	2.45 (0.014)*
	3	6	78.1% (0.001)	−1.794 (−2.513∼−1.075)	4.89 (0.001)***
D	1	3	85.6% (0.001)	−0.569 (−0.924∼−0.214)	1.76 (0.079)
	2	3	13.9% (0.313)	−0.280 (−0.497∼−0.063)	2.53 (0.012)*
	3	8	72.7% (0.001)	−1.420 (−1.860∼−0.980)	3.50 (0.001)***
A	1	2	36.9% (0.208)	−0.331 (−1.273∼0.611)	0.69 (0.491)
	2	3	70.9% (0.032)	−0.367 (−0.728∼−0.006)	1.99 (0.046)*
	3	8	83.2% (0.001)	−1.674 (−2.492∼−0.855)	4.01 (0.001)***
F	1	3	86.5% (0.001)	−0.769 (−1.616∼0.078)	1.78 (0.075)
	2	3	71.0% (0.032)	−0.131 (−0.756∼0.495)	0.41 (0.682)
	3	8	84.2% (0.001)	−2.129 (−3.058∼−1.200)	4.49 (0.001)***
C	1	2	72.4% (0.027)	−0.843 (−1.163∼−0.523)	5.16 (0.001)***
	2	3	0.0% (0.476)	−0.239 (−0.463∼−0.015)	2.09 (0.037)
	3	6	82.8% (0.001)	−1.181 (−1.922∼−0.440)	3.12 (0.002)**
V	1	3	93.0% (0.001)	1.881 (0.265∼3.498)	2.28 (0.023)*
	2	3	0.0% (0.691)	0.091 (−0.227∼0.409)	0.56 (0.577)
	3	8	65.1% (0.005)	1.996 (1.408∼2.584)	6.65 (0.001)***

T, tension and anxiety subscale; D, depression subscale; A, anger and hostility subscale; F, fatigue subscale; C, confusion subscale; V, vigor subscale.

*p < 0.05; **p < 0.01; ***p < 0.001.

(2) Subgroup analysis of intervention types

The results of the subgroup analysis and heterogeneity test based on different physical activity patterns are presented in Table 4. Using the T-scale score as the outcome variable, the combined effect size for subgroup 1 indicated that the experimental group showed lower tension-anxiety mood compared to the control group, but the effect size was not significant (*Z* = 1.85, *P* = 0.064). In subgroup 2, the experimental group exhibited significantly lower tension-anxiety mood than the control group (*Z* = 3.90, *P* = 0.000), with a large effect size (WMD = −1.454). Additionally, the combined effect sizes for the D, A, F, C, and V subscales as outcome indicators all suggested that the experimental group demonstrated greater mood improvement. The effect sizes for both subgroup 1 and subgroup 2 were significant (*P* < 0.05). However, the absolute effect sizes for the dynamic subgroup were generally larger than those for the static subgroup, with a higher significance level (*P* < 0.01). Based on these findings, dynamic forest therapy activities are more effective in improving both positive and negative psychological states than static forest therapy activities, with specific effect size levels presented in Table 4.

**TABLE 4:** Subgroup analysis of intervention types.

Outcome indicators	Subgroup 1: (Static) 2: (Dynamic)	Quantity	Heterogeneity test	Effect size	
			I^2^% (*P*)	WMD (95% CI)	Z (*P*)
T	1	4	85.4% (0.001)	−0.973 (−2.004∼−0.058)	1.85 (0.064)
	2	7	91.6% (0.001)	−1.454 (−2.185∼−0.723)	3.90 (0.001)***
D	1	5	86.4% (0.001)	−0.599 (−1.095∼−0.103)	2.37 (0.018)*
	2	9	72.1% (0.001)	−0.626 (−1.010∼−0.242)	3.20 (0.001)***
A	1	4	92.5% (0.001)	−1.192 (−2.204∼−0.180)	2.31 (0.021)*
	2	9	88.4% (0.001)	−0.026 (−1.647∼−0.405)	3.24 (0.001)***
F	1	5	88.1% (0.001)	−1.161 (−1.922∼−0.401)	2.99 (0.003)**
	2	9	91.7% (0.001)	−1.429 (−2.452∼−0.407)	2.74 (0.006)**
C	1	4	75.0% (0.003)	−0.628 (−0.971∼−0.284)	3.58 (0.001)***
	2	7	91.0% (0.001)	−1.347 (−2.044∼−0.651)	3.79 (0.001)***
V	1	5	77.1% (0.002)	0.841 (0.139∼1.488)	2.36 (0.018)*
	2	9	92.1% (0.001)	1.728 (0.764∼2.691)	3.51 (0.001)***

T, tension and anxiety subscale; D, depression subscale; A, anger and hostility subscale; F, fatigue subscale; C, confusion subscale; V, vigor subscale.

*p < 0.05; **p < 0.01; ***p < 0.001.

(3) Subgroup analysis of subject sources

The results of the subgroup analysis and heterogeneity test based on participants’ ethnic origins are presented in Table 5. Using the T-scale score as the outcome variable, the combined effect size for subgroup 1 showed that the experimental group had a significant reduction in tension-anxiety mood compared to the control group (*Z* = 4.20, *P* = 0.000), with a large effect size (WMD = −1.772). For subgroup 2, the experimental group also showed a significant reduction in tension-anxiety mood compared to the control group (*Z* = 2.45, *P* = 0.014), with a moderate effect size (WMD = −0.458). Additionally, using the D, A, F, C, and V subscale scores as outcome indicators, the combined effect sizes for the Asian subgroup indicated a significantly better mood improvement in the experimental group (*P* < 0.001). In contrast, for the European subgroup, the combined results for the A, F, and V scores showed improved mood in the experimental group, but the effect size was not significant (*P* > 0.05). The results suggest that the effects of forest therapy appeared stronger in the Asian samples for both negative emotions (e.g., tension, depression, confusion) and positive emotions (e.g., vitality). It is important to note, however, that cultural, contextual, or methodological factors may have contributed to this observed difference, with specific effect size levels presented in Table 5.

**TABLE 5:** Subgroup analysis of subject sources.

Outcome indicators	Subgroup 1: (Asian) 2: (European)	Quantity	Heterogeneity test	Effect size	
			I^2^% (*P*)	WMD (95% CI)	Z (*P*)
T	1	8	87.5% (0.001)	−1.772 (−2.599∼−0.945)	4.20 (0.001)***
	2	3	67.6% (0.046)	−0.458 (−0.824∼−0.092)	2.45 (0.014)*
D	1	8	71.4% (0.001)	−0.889 (−1.262∼−0.515)	4.66 (0.001)***
	2	6	18.9% (0.290)	−0.227 (−0.407∼−0.047)	2.47 (0.014)*
A	1	8	93.7% (0.001)	−1.455 (−2.309∼−0.602)	3.34 (0.001)***
	2	5	69.8% (0.010)	−0.439 (−0.887∼0.09)	1.92 (0.055)
F	1	8	90.6% (0.001)	−2.050 (−2.911∼−1.189)	4.67 (0.001)***
	2	6	60.7% (0.026)	−0.215 (−0.698∼0.267)	0.87 (0.382)
C	1	8	84.9% (0.001)	−1.268 (−1.882∼−0.655)	4.05 (0.001)***
	2	3	0.0% (0.476)	−0.239 (−0.463∼−0.015)	2.09 (0.037)*
V	1	8	81.2% (0.001)	2.053 (1.391∼2.714)	6.08 (0.001)***
	2	6	3.6% (0.394)	0.184 (−0.058∼0.427)	1.49 (0.137)

T, tension and anxiety subscale; D, depression subscale; A, anger and hostility subscale; F, fatigue subscale; C, confusion subscale; V, vigor subscale.

*p < 0.05; ***p < 0.001.

(4) Literature bias test

Due to the subjectivity of the funnel plot test, a quantitative method was employed to assess publication bias. The results indicated that the meta-analysis using the T-scale and V-scale scores as outcome indicators was unstable (PT = 0.000, *P* < 0.05; PV = 0.001, *P* < 0.05), suggesting the presence of publication bias. No publication bias was found in the meta-analyses using scores from other subscales as outcome indicators (*P* > 0.05) (Table 6).

**TABLE 6:** Literature bias test.

	T	D	A
*P*-value	0.001	0.636	0.615
	F	C	V
*P*-value	0.573	0.757	0.001

## 4 Discussion

Forest therapy is a non-pharmacological intervention that effectively improves mental health. This study conducted a meta-analysis using the POMS score as the outcome measure to examine the impact of forest therapy on mood states. The six subscales of the POMS, which represent anxiety, depression, anger, fatigue, confusion, and vitality, were analyzed, and subgroup analysis was performed based on intervention duration, exercise intensity, and participant source. The subgroup analysis results showed that forest therapy lasting 15 min had a positive but insignificant effect on reducing anxiety, depression, and anger, as well as improving vitality. In contrast, forest therapy lasting 30 and 60 min had a significant positive impact. This supports the potential existence of a dose-response relationship in nature exposure. Longer durations may provide individuals with sufficient time to disengage from orienting fatigue and progress through the four stages described by the Attention Restoration Theory—distancing, extension, enchantment, and compatibility—thereby achieving deeper psychological recovery. Regarding anxiety reduction, static forest therapy showed no significant effect, while both static and dynamic forest therapies significantly improved other emotional indicators. For reducing anger, fatigue, and increasing vitality, forest therapy was more effective in Asian participants than in European participants, with significant improvements observed in the former group. This result is now discussed as strong support for the synergistic effect central to the “Green Exercise” framework. We posit that dynamic activities in a forest environment combine the established psychological and physiological benefits of physical activity with the restorative properties of nature, potentially creating an effect greater than the sum of its parts. This is contrasted with the more passive restoration potentially offered by static therapy, which aligns more closely with Stress Reduction Theory (SRT).

(1) Key finding

The benefits of forest therapy are directly reflected in the improvement of mental health. Several studies have reported the differences between the experimental and control groups in terms of mental health indicators. The most commonly used mental health indicator is emotional state, which is often measured using the Profile of Mood States (POMS). The results indicated that forest therapy produced positive changes in multiple mental health indicators compared to the control group. However, not all studies reported similar changes in indicators. Horiuchi et al. (2014, 2015) found that, for POMS score data, the main effect of time was significant, but the main effect of group was not, regardless of whether participants were in the closed or forest viewing condition. Additionally, the subscale scores for anxiety (T-A), depression (D), fatigue (F), and confusion (C) decreased significantly following forest viewing, while the scores for anger-hostility (A-H) and vitality (V) did not change significantly. Notably, the two intervention conditions in this study differed by only 30 min (Horiuchi et al., 2014). Sonntag-Öström et al. (2015) conducted forest therapy activities twice a week for one year, and the results showed that the experimental conditions effectively improved job burnout, self-esteem, and depression levels among participants, although the differences compared to the control group were not statistically significant. Gidlow et al. (2016) compared the effects of walking in forest, lakeside, and urban environments and found that, in all environments, the main effect of time on TMD scores was significant (*P* = 0.009), but the main effect of group was not significant (*P* = 0.178). Lee et al. (2011) used a paired sample *t*-test to examine pre- and post-test differences within the group, but did not report inter-group difference results. The results showed that after a 3-day forest therapy program, stress, depression, anger, fatigue, and confusion were significantly reduced, and vitality increased. In contrast, urban programs significantly reduced stress, but did not alleviate depression, anger, fatigue, or confusion, nor did they increase vitality, highlighting the mental health benefits of forest therapy.

Compared to scale-based assessment methods, physiological indicators are more objective and provide a clearer reflection of both physical and mental health levels. Moreover, physiological indicators are closely linked to mental health markers, making them commonly used to assess the benefits of forest therapy. A decrease in systolic or diastolic blood pressure can indicate alleviation of stress, anxiety, and other adverse mental conditions (Zijlema et al., 2018). In time-domain analysis of heart rate variability, a decrease in the SDNN and RMSSD indexes reflects a reduction in parasympathetic nerve activity. In frequency-domain analysis, an increase in the LF/HF ratio signals enhanced nerve tone, with decreased parasympathetic and increased sympathetic nerve activity linked to improved positive emotions (Sin-Ae et al., 2017). Additionally, epinephrine and salivary cortisol serve as biomarkers of stress (Lee et al., 2011; Li et al., 2011), brain electrical indicators, such as the alpha and beta waves, also reflect psychological changes. The alpha wave is typically associated with the brain’s electrical activity during calm, awake states, while the beta wave is linked to tense and excited states of the cerebral cortex (Ahmad et al., 2018). Therefore, physiological indicators and subjective mental health assessments often complement each other in studies on forest therapy interventions.

The included studies reported varying conclusions regarding changes in blood pressure indices. Some studies found significant differences in blood pressure changes between the forest therapy group and the control group (*P* < 0.05) (Janeczko et al., 2020; Lee et al., 2014; Liu et al., 2021; Li et al., 2011). However, some results indicated no significant difference in blood pressure between the groups (*P* > 0.05) (Song et al., 2019; Wen et al., 2023). Ahmad et al. (2018) investigated the performance of alpha and beta brain waves in two different environments and found that participants’ 15-min average alpha waves increased significantly after walking in a bamboo forest. Regarding the regulation of hormones and blood parameters by forest therapy, Genxiang et al. (2017) observed that cardiovascular disease-related biomarkers, including endothelin (ET-1), renin, angiotensinogen (AGT), angiotensin (ANGII), and ANGII receptor types 1 and 2, were lower in participants exposed to a forest environment compared to urban controls. Masahiro et al. (2015) reported that forest viewing decreased cerebral oxygenated hemoglobin (HbO2) levels in participants. Lee et al. (2011) found that salivary cortisol levels decreased significantly in the forest therapy group compared to the urban environment. Additionally, Lee et al. (2014) showed that forest therapy significantly improved lung function, with increases in FEV1 (*P* < 0.01) and FEV6 (*P* < 0.01). Overall, the forest therapy intervention has demonstrated positive and beneficial effects on the regulation of physiological and biochemical markers.

(2) Clinical significance

Studies have demonstrated that the effects of forest therapy can be influenced by various covariates and confounding factors. Guan et al. (2017) conducted a field-controlled study on the healing effects of different forest systems in Changchun’s Central Park, China. Their comparison revealed that participants in the maple forest group experienced a reduction in learning-related anxiety, while those in the birch forest group showed the greatest reduction in employment stress anxiety. Additionally, participants in the oak forest reported higher levels of anxiety than those in the birch forest. Besides tree species, the forest composition also appears to influence mental health improvements. Liu et al. (2021) found that, compared to broadleaf and coniferous forests, mixed forests were more effective in reducing blood pressure and heart rate while increasing vitality. Recovery and positive mental health levels were significantly higher in the coniferous forest, while all subscales of the Profile of Mood States (POMS), except for vitality, showed significant reductions.

Seasonal variations also affect health improvements. Katja et al. (2022) observed that participants in the forest group showed significant changes in subjective self-perception, with notable differences in the total score (*P* = 0.054) and sub-items (*P* = 0.028). These effects were more pronounced in summer, with no correlation found in autumn. Short-term stays in the forest during the summer resulted in greater improvements in mood and wellbeing compared to those in the wild, an effect that was not observed in the fall. Chen et al. (2018) showed that the emotional regulation effect of forest therapy was more pronounced in suburban development zones during spring than in semi-primitive forest parks (*P* = 0.07). In contrast, the emotional regulation effect was stronger in semi-primitive areas during summer and autumn compared to suburban forest parks (*P* = 0.062 in summer, *P* = 0.001 in autumn), with the emotional improvement effect of different forest types showing seasonal specificity. Furuyashiki et al. (2019) discussed the impact of forest therapy on improving mental health in individuals with varying levels of depression. Of the 155 participants, 37% were prone to depression, and all participants showed significant reductions in diastolic blood pressure and negative mood after forest therapy, with depression-prone individuals showing significantly higher improvements on many POMS subscale items than those without depressive tendencies.

## 5 Conclusion

This study provides evidence that forest therapy is associated with reduced negative emotional states and increased vitality levels in adult populations. However, several important limitations must be acknowledged when interpreting these findings: The primary limitation is the geographic imbalance in the existing literature, as our analysis sample primarily drew from studies in Asia. This restricts the generalizability of results to other cultural and geographic contexts and suggests that cultural factors or specific forest types may influence intervention outcomes. Furthermore, treatment effects appear to be moderated by intervention design—interventions lasting over one hour and incorporating dynamic activities demonstrated more consistent outcomes. Future research should validate these findings across more diverse populations. Despite these limitations, current evidence suggests forest therapy holds promise as a non-pharmacological adjunct for promoting mental health. Practice recommendations emphasize implementing interventions of sufficient duration that integrate active components.

## 6 Research prospect

Following the identification of the positive effects of forest therapy on both physical and mental health, this study aims to further explore the factors influencing these benefits. The study of ecological exposure and its role in sports health promotion involves many potential confounding variables, and the significance of forest therapy as a holistic reflection of both ecological exposure and sports health promotion should not be underestimated. In terms of ecological exposure, the study highlighted the significant effects of forest composition (including coniferous, deciduous, and mixed forests) and seasonal variations (spring, summer, and autumn) on therapeutic outcomes. Regarding physical activity, in addition to the intervention duration emphasized in this study, significant differences were observed in the effects of varying exercise intensities during forest therapy. Further investigation and identification of additional moderating variables will aid in optimizing forest therapy programs and enhancing their practical value.

## Data Availability

The original contributions presented in this study are included in this article/supplementary material, further inquiries can be directed to the corresponding author.
